# Influence of electrode array stiffness and diameter on hearing in cochlear implanted guinea pig

**DOI:** 10.1371/journal.pone.0183674

**Published:** 2017-08-24

**Authors:** Mylène Drouillard, Renato Torres, Elisabeth Mamelle, Daniele De Seta, Olivier Sterkers, Evelyne Ferrary, Yann Nguyen

**Affiliations:** 1 Sorbonne Universités, Université Pierre et Marie Curie Paris 6, Inserm, Unité “Réhabilitation chirurgicale mini-invasive et robotisée de l'audition”, Paris, France; 2 AP-HP, GHU Pitié-Salpêtrière, Service ORL, Otologie, implants auditifs et chirurgie de la base du crâne, Paris, France; The University of Sydney, AUSTRALIA

## Abstract

During cochlear implantation, electrode array translocation and trauma should be avoided to preserve residual hearing. The aim of our study was to evaluate the effect of physical parameters of the array on residual hearing and cochlear structures during insertion. Three array prototypes with different stiffnesses or external diameters were implanted in normal hearing guinea pigs *via* a motorized insertion tool carried on a robot-based arm, and insertion forces were recorded. Array prototypes 0.4 and 0.4R had 0.4 mm external diameter and prototype 0.3 had 0.3 mm external diameter. The axial stiffness was set to 1 for the 0.4 prototype and the stiffnesses of the 0.4R and 0.3 prototypes were calculated from this as 6.8 and 0.8 (relative units), respectively. Hearing was assessed preoperatively by the auditory brainstem response (ABR), and then at day 7 and day 30 post-implantation. A study of the macroscopic anatomy was performed on cochleae harvested at day 30 to examine the *scala* location of the array. At day 7, guinea pigs implanted with the 0.4R array had significantly poorer hearing results than those implanted with the 0.3 array (26±17.7, 44±23.4, 33±20.5 dB, n = 7, *vs* 5±8.7, 1±11.6, 12±11.5 dB, n = 6, mean±SEM, respectively, at 8, 16 and 24 kHz, p<0.01) or those implanted with the 0.4 array (44±23.4 dB, n = 7, *vs* 28±21.7 dB, n = 7, at 16 kHz, p<0.05). Hearing remained stable from day 7 to day 30. The maximal peak of insertion force was higher with the 0.4R array than with the 0.3 array (56±23.8 mN, n = 7, vs 26±8.7 mN, n = 6). Observation of the cochleae showed that an incorrectly positioned electrode array or fibrosis were associated with hearing loss ≥40 dB (at 16 kHz). An optimal position in the *scala tympani* with a flexible and thin array and prevention of fibrosis should be the primary objectives to preserve hearing during cochlear implantation.

## Introduction

Initial indications for cochlear implantation have shifted over time from patients with complete bilateral deafness, to include patients with residual hearing at lower frequencies but who have limited understanding performance with hearing aids. Ten to thirty percent of patients lose useful residual hearing with acoustic amplification after cochlear implantation [1,2]. This residual hearing at low frequencies would improve hearing in noisy environments and perception of musical melodies when electric acoustic stimulation is used for hearing rehabilitation. Different strategies have been developed to preserve this residual hearing, the most important of which is to decrease mechanical trauma during array insertion and to prevent basilar membrane rupture, osseous spiral lamina fracture, and electrode translocation from the *scala tympani* to the *scala vestibuli* [3,4]. Technical refinements of the surgical technique, which is currently performed manually, have been adopted with the “soft surgery” technique. Local drug delivery (Dexamethasone, N-acetyl-cysteine, BDNF, Cytarabine) to achieve otoprotection has also been proposed [5,6]. In addition, array design with a thinner and softer electrode is an increasing trend. Stiffness and diameter of the array appear to be determining factors in decreasing frictional forces and cochlea trauma, but they are not often studied as two separate factors [1,7]. A better understanding of the influence of these parameters on surgical outcomes was the first objective of this study.

Another line of research to improve residual hearing is to monitor and eventually control frictional forces during electrode array insertion. For example, it has been shown that the use of a motorized insertion tool could change the insertion force profile with less stops and starts compared to manual insertion [8] and this approach has enhanced preservation of residual hearing in an animal model [9]. Moreover, some authors have suggested including a force sensor placed at the tip of the electrode array to detect contact with the lateral wall or basilar membrane [10]. However, the existence of a relationship between electrode array insertion force and post-implantation hearing outcomes remains to be demonstrated. This was the secondary objective of this study.

## Materials and methods

### Animals

Twenty normal hearing male Hartley albino guinea pigs (Charles River France, Domaine des Oncins, L’Arbresle, France), aged from 3 to 5 months, weighing 372 ± 22.7 g (mean ± SD) were included in this study. After obtaining the guinea pigs, the animals were given a week to acclimate to their environment. They were housed two animals to each cage with sawdust as bedding material in temperature (22–25°C) conditions and light/dark cycle (12/12 h) controlled rooms. The animals had access to food and water ad libitum. All animals were closely monitored to look for behaviors indicating excessive pain or infection. None of these behaviors was observed during the duration of the study. All experiments were conducted in accordance with the guidelines established by the European Communities Council Directive (2010/63/EU Council Directive Decree), which are similar to those described in the Society of Neuroscience guidelines for the Use of Animals in Neuroscience Research. All experiments were conducted in line with these regulations and the protocol was prospectively approved by the ethics committee n°121 “Bichat-Debré-St Louis” (agreement N°2016–040717097444).

### Electrode array

The electrode arrays (Oticon medical, Vallauris, France) were silicone prototypes with six platinum iridium electrodes designed for use in guinea pigs. Three different types of 8 mm long electrode array were used (0.4R, 0.4, 0.3) with different external diameters (0.3 or 0.4 mm) or different axial stiffnesses (Table 1). The axial stiffness depends on the configuration of the wires connected to the electrodes. It was calculated by horizontal deflection measured by Oticon medical, and was arbitrarily set to 1 for the 0.4 electrode array (relative unit). This array was set as the “standard” for guinea pigs. The stiffnesses of the two other arrays were calculated from this standard value (Table 1).

**Table 1:** Electrode array prototypes inserted in guinea pig cochleae in vivo.

Prototype array	External diameter (mm)	Inserted length (mm)	Electrode array configuration	Stiffness*
0.4	0.4	4	6 wires Ø 0.25 μm	1
0.4R	0.4	4	5 wires Ø 0.25 μm + 1 wire Ø 0.5 μm	6.8
0.3	0.3	4	6 wires Ø 0.25 μm	0.8

* Axial stiffness was calculated by Oticon medical; the stiffness of the 0.4 array was set to 1 (relative unit), and the stiffnesses of the two other arrays were calculated from this value.

### Study design

Seven guinea pigs were implanted with the 0.4 electrode array, seven with the 0.4R electrode array and six with the 0.3 electrode array. A linear motorized electrode array insertion tool [8] carried by a robotic arm [11] was used to insert the arrays with a constant insertion speed of 0.25 mm/s. The insertion was performed until four electrodes had been placed in the cochlea to ensure a reproducible insertion length (4 mm). Hearing was measured by auditory brainstem response (ABR), 5 days before implantation, at day 7 (D7) and day 30 (D30) after implantation. After sacrifice at D30, the macroscopic anatomy of the cochlea was examined with the electrode array in place, in 12 animals selected according to their post-implantation hearing threshold (6 animals with the best hearing and 6 animals with the worst hearing).

### Surgery

In order to avoid any transcranial bone conduction from one ear to the other that could have affected the ABR measurements, the cochlea was damaged on one side by breaking the modiolus with a 1 mm diamond burr. The large access hole drilled into the damaged cochlea was then sealed with temporal muscle to avoid any perilymph leakage.

Implantation surgery was performed under aseptic conditions. General anesthesia was administered by an intramuscular injection of a combination of 4 mg/kg Xylazine (Bayer, Puteaux, France), and 60 mg/kg Ketamine (Virbac, Carros, France) along with a post-auricular injection of 1% Xylocaine (0.5 ml sc, Aguettant, Lyon, France). After a post-auricular incision, the muscles were carefully dissected to expose the bulla. The bulla was opened with a 2 mm round fluted burr (Primado 2, NSK, Tochighi-Ken, Japan). The cochlea was thus visualized, and a cochleostomy was gradually performed with 0.3 to 0.6 mm trephines for the 0.4 mm diameter electrode arrays, and with 0.3 to 0.5 mm trephines for the 0.3 mm diameter electrode array. Hyaluronic acid (Healon 10 mg/ml, Albott, Uppsala, Sweden) was applied to the cochleostomy. The guinea pig was then settled in a hammock with its head fixed in a frame placed on a 6-axis sensor (ATI, Nano 17, calibration type SI-12-0.12, resolution: 3 mN, Apex, NC, USA). The hammock and frame were designed to decrease respiratory artefacts during measurement of insertion forces. The electrode array was loaded in the insertion tool [8] which was held by a robotic arm [11] and was inserted at a constant speed of 0.25 mm/s. The insertion site was sealed with small autologous muscle grafts. Muscle and skin were sutured with separate stitches of Vicryl 3.0.

An analgesic (Ibuprofen, 10 mg/kg po, Pfizer, Paris, France) and an antibiotic (Enrofloxacine, 10 mg/kg im, Bayer, Puteaux, France) were administered when the animal woke up and on the day after surgery.

### ABR measurements

The hearing thresholds of ABR measurements were recorded (Otophylab, RT-Conception Ltd., Clermont-Ferrand, France) under general anesthesia (Xylazine and Ketamine), 5 days before implantation, and 7 (D7) and 30 days (D30) after implantation. Before ABR measurement, eardrum integrity and lack of cerumen impaction were checked by otoscopy. The animals were stimulated *via* a calibrated tube in the external auditory canal with a click of 100 μs duration and by acoustic tone burst stimuli at 2000, 4000, 8000, 16000, 24000 and 32000 Hz from 90 dB to the threshold with decreasing intensity in 5 dB steps. Each stimulus was presented 500 times. Masking was not necessary since the contralateral cochlea had been surgically deafened. The collecting electrode needles were positioned on the vertex (reference electrode), on the mastoid of the implanted ear (measurement electrode) and on the left hind leg (ground electrode). The hearing threshold of the animal was determined by the lowest intensity which evoked a visually replicable (three times) waveform. The measurement resolution was ± 5 dB.

### Force measurement

A 6-axis force sensor (ATI Nano 17^®^, calibration type SI-12-0.12, resolution 1/320, Apex, NC, USA) was used to measure the insertion forces. Only the orthogonal components Dx, Dy, and Dz, provided by the 6-axis force sensor, were averaged to obtain the norm of the force applied to the cochlea. The sensor was fixed under the frame where the animal’s head was fixed. During the insertion, particular attention was addressed to avoid any contact of the robot-based arm and the insertion tool with the animal. Only the array ejected from the insertion tool would touch the animal while penetrating into the cochlea. This allowed measurement of electrode array friction force in the cochlea. Three parameters were analyzed from the data collected by the force sensor: the maximal peak of insertion force, the momentum and jerk [8]. The maximum force can be interpreted as the peak force that can lead to maximal damage to the cochlea. The total change in momentum represents the global force that is applied to achieve complete insertion. Jerk represents rapid variation of insertion force caused by, for example, tremor during insertion.

### Macroscopic anatomy

**Fig 1:**
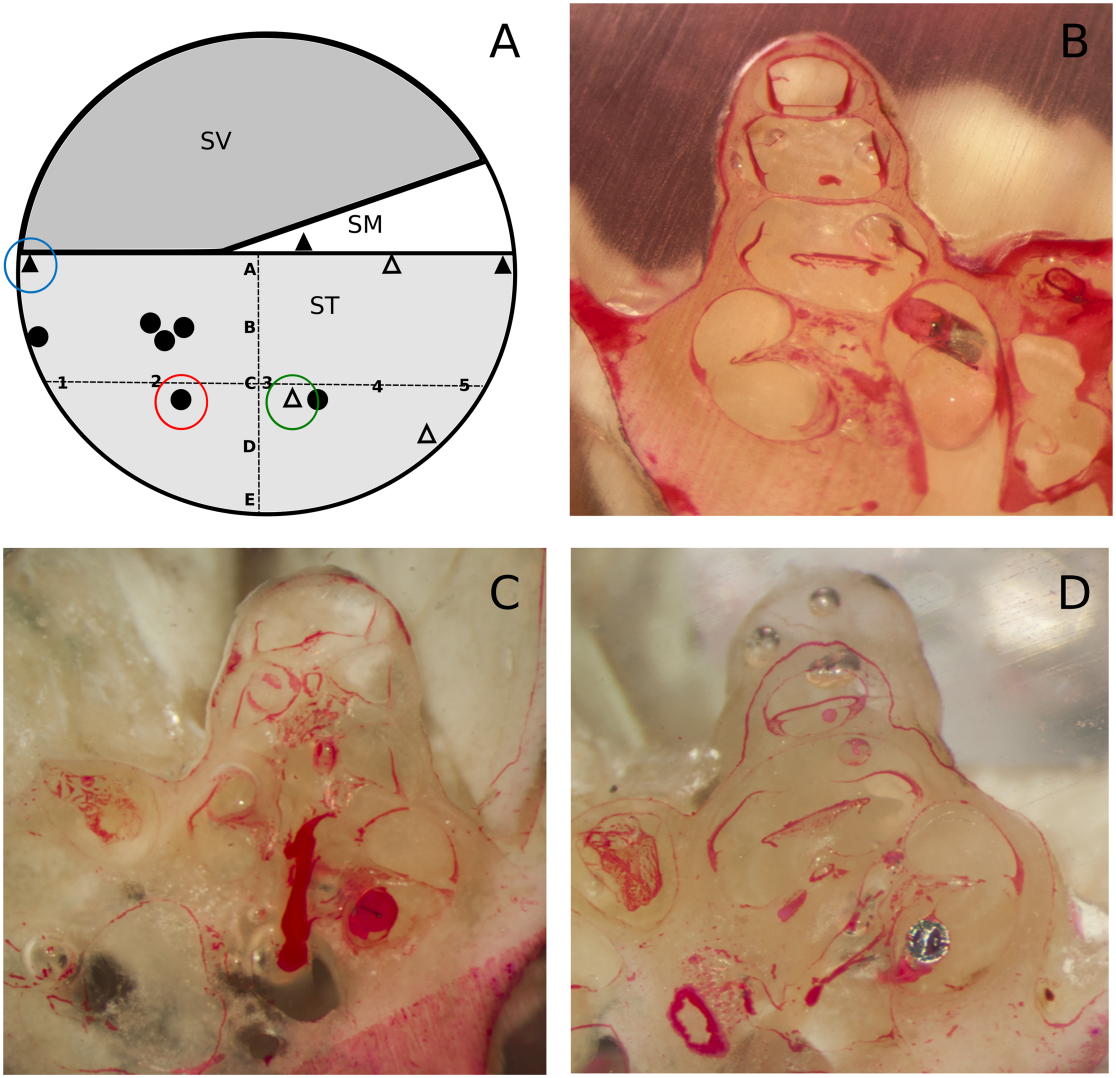
Electrode array placement in the cochlea. (A) Diagram of a cochlea section passing through the three scala (SV, scala vestibuli; SM, scala media; ST, scala tympani) with a classification according to the placement of electrodes. From A (close to the basilar membrane) to E in a vertical orientation, and from 1 (close to the modiolus) to 5 (close to the lateral wall) in a horizontal orientation. Black circles correspond to electrode placement without hearing loss at D30, black triangles correspond to electrode placement with hearing threshold shift >40 dB at D30 without fibrosis, and light grey triangles correspond to fibrosis. (B) Macroscopic anatomy section of an electrode array in A placement, in contact with a fracture of the osseous spiral lamina (blue circle in A). (C) Macroscopic anatomy section of an electrode array in 4C placement surrounded by reaction fibrosis * (green circle in A). (D) Macroscopic anatomy section of an electrode array in 2C placement away from basilar membrane and osseous spiral lamina (red circle in A).

At D30, the guinea pigs were sacrificed with a lethal trans-cardiac injection of 3 ml of sodium pentobarbital (54.7 mg/ml, Ceva Santé Animale, Libourne, France). The cochleae were harvested with the electrode array in place. The cochlear apex was then carefully opened. At that point, the cochleae were fixed with formalin (Formol 4%, VWR Chemicals, Fontenay-sous-Bois, France) over a 48 h period, dehydrated in ethanol (70°, 90°, 95°) over a 3 h period for each, soaked in a 50/50 solution of acetone and ethanol 100°, and finally embedded in Crystal resin (Gedeo, Pebeo, Gemenos, France). The embedded cochleae were manually ground with progressive sandpaper (120, 320, 600, 7000). The polished surface was stained with a 3% aqueous solution of Phloxin B (RAL diagnostics, Martillac, France) for 5 min, rinsed and photographed using an ISM-2 Kaps microscope system (Karl Kaps GmbH&Co. KG, Asslar/Wetzlar, Germany) at increasing magnifications (0.63, 1, 1.6, focal length 200 mm). Pictures were taken at each electrode, and the array location, the presence of fibrosis and the integrity of the structures (osseous spiral lamina, basilar membrane) were analyzed according to the Eshraghi trauma scale [12]. A classification scheme for the electrode array position in the cochlea was designed, with an alphabetic scale in the vertical orientation of A (in contact with the basilar membrane) to E, and a numerical scale in the horizontal orientation of 1 (in contact with the modiolus) to 5 (in contact with the lateral wall) (Fig 1A). The analysis of macroscopic anatomy photographs was performed by an observer who was not involved with the surgical procedure, and who was not aware of the array type or the post-implantation hearing. This study on the macroscopic anatomy was performed on 12 animals selected according to their post-implantation hearing threshold (6 animals with the best hearing and 6 animals with the worst hearing). Semi-quantitative information was reported on the presence of fibrosis in the basal turn (+ less than 10%, ++ 10–50%, +++ over 80% of the scala tympani lumen invaded by fibrosis). Even though, it would have raised the strength of the analysis of the study, the macroscopic anatomy could not be performed in all animals due to technical issues.

### Data analysis

All statistical analysis was performed with Prism software (GraphPad Software, Inc., La Jolla, CA, USA). Values are presented as means ± SEM. Comparisons were done with one-way or two-way Anova with subsequent post-hoc Tukey test. Assessment of a correlation between insertion forces and hearing threshold shift was performed with Pearson’s linear regression tests. A p value < 0.05 was considered to be statistically significant.

## Results

### Hearing loss after cochlear implantation

**Fig 2:**
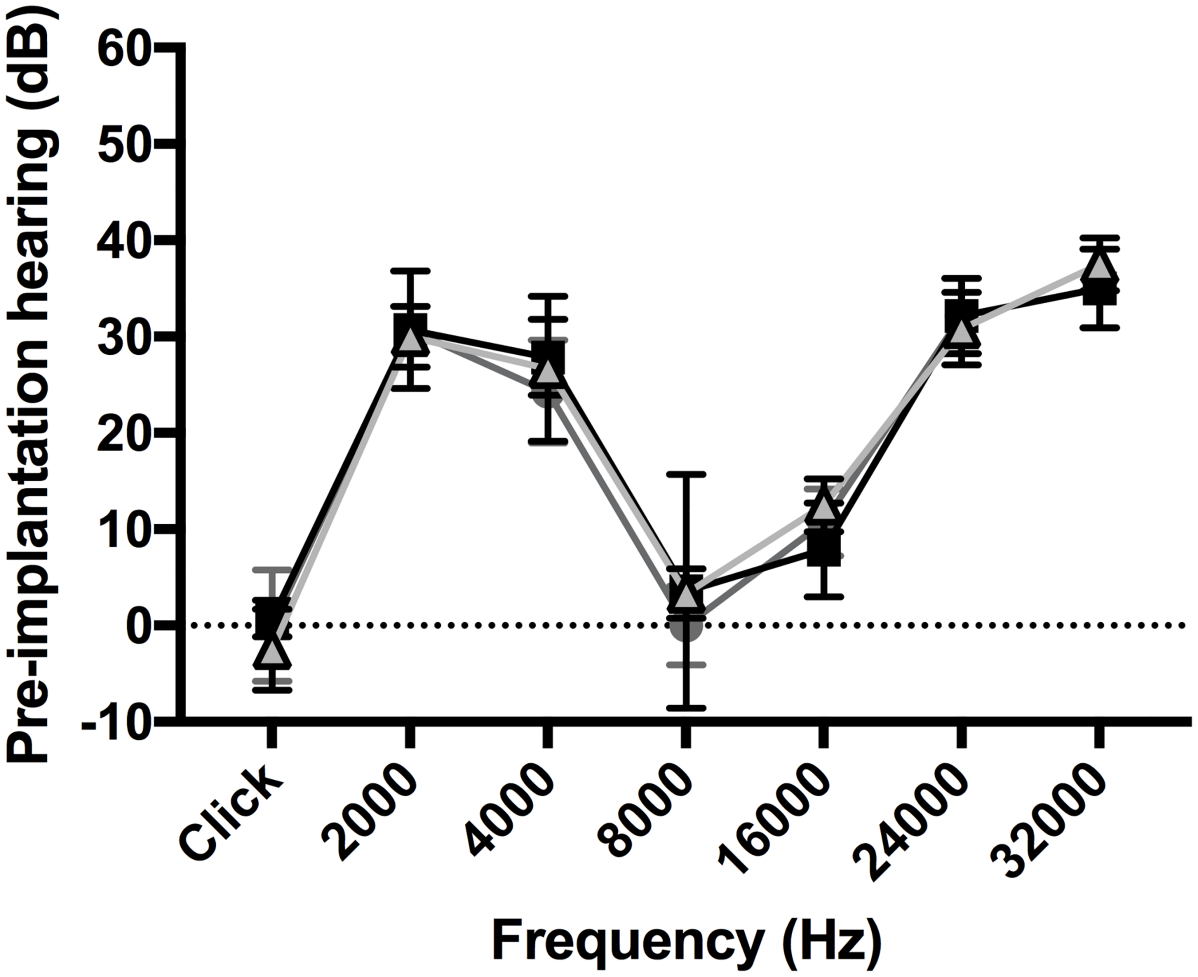
Pre-implantation hearing (dB) according to the electrode array. Preoperative measurement according to the animal group: 0.4R (black squares) (n = 7), 0.4 (dark grey circles) (n = 7), and 0.3 (light grey triangles) (n = 6). Before implantation, the animals in the three groups had similar hearing thresholds (dB).

Before implantation, the animals in the three groups had similar hearing thresholds (dB) (Fig 2).

**Fig 3:**
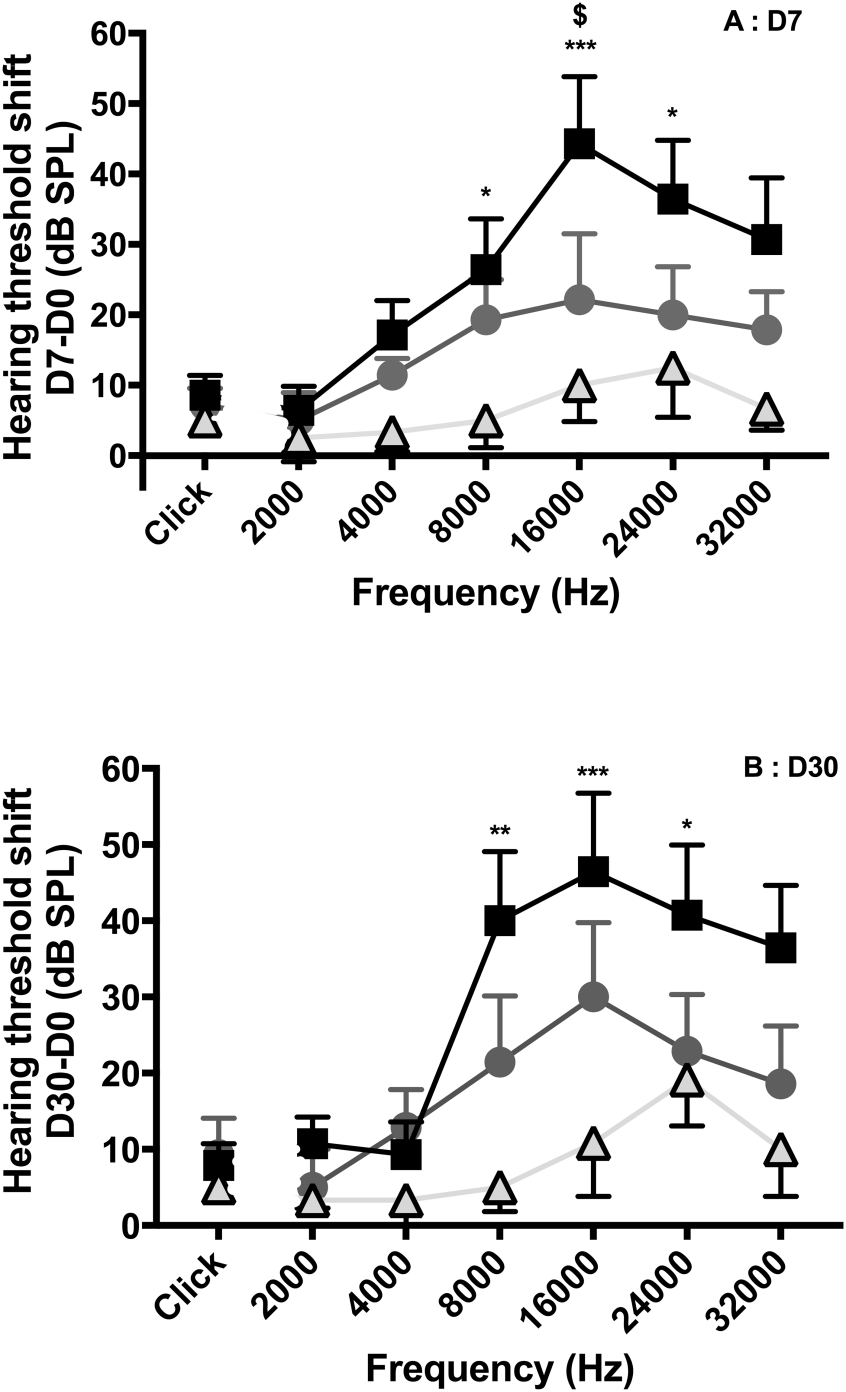
Hearing loss (dB) between D7 and D30 according to the electrode arrays. Hearing threshold shift between D7 and D30 measurement according to the animal group: 0.4R (black squares) (n = 7), 0.4 (dark grey circles) (n = 7), and 0.3 (light grey triangles) (n = 6). At D30, hearing loss was unchanged compared to that at D7, and this applied to each group.

At D7 after implantation, hearing loss was observed in the three groups, predominantly at 8000, 16000 and 24000 Hz. A significant difference was observed between the three groups (two-way Anova, p<0.01), with more significant hearing loss with the 0.4R array than with the 0.3 array, and intermediate results with the 0.4 array (Fig 3A). Significant differences were noted between the 0.4R and 0.3 arrays at 8000 Hz (26 ± 17.7 dB, n = 7, *vs* 5 ± 8.7 dB, n = 6, p<0.05), at 16000 Hz (44 ± 23.4 dB, n = 7, *vs* 10 ± 11.6 dB, n = 6, p<0.001) and at 24000 Hz (33 ± 20.5 dB, n = 7, *vs* 12 ± 11.5 dB, n = 6, p<0.05). Significant differences were also noted between the 0.4 and 0.4R arrays at 16000 Hz (44 ± 23.4 dB, n = 7, *vs* 28 ± 21.7 dB, n = 7, p<0.05).

### Hearing loss evolution between D7 and D30

**Fig 4:**
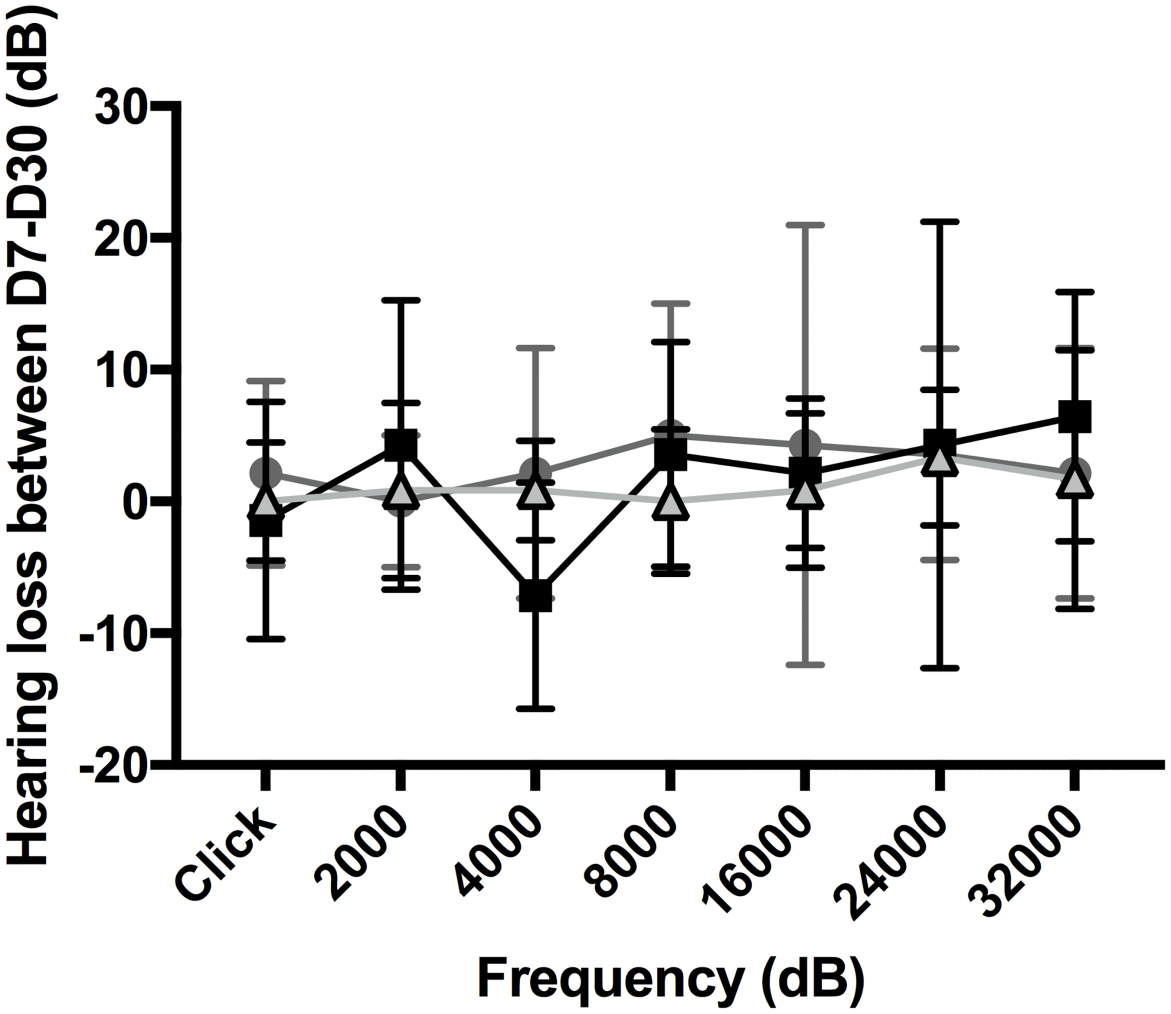
Influence of electrode array geometry on hearing loss. A: D7. B: D30. Hearing threshold shift measurement according to 0.4R (black squares) (n = 7), 0.4 (dark grey circles) (n = 7), and 0.3 (light grey triangles) (n = 6) electrode arrays, inserted at 0.25 mm/s. Values are means ± SEM. Statistical analysis was with two-way Anova (p<0.001 at D7, and p<0.05 at D30), comparison between 0.3 and 0.4R (*p<0.05, **p<0.01, ***p<0.001), comparison between 0.4 and 0.4R (^$^p<0.05).

At D30, hearing loss was unchanged compared to that at D7, and this applied to each group (Figs 3B and 4).

### Relationship between insertion forces and hearing loss after cochlear implantation

**Fig 5:**
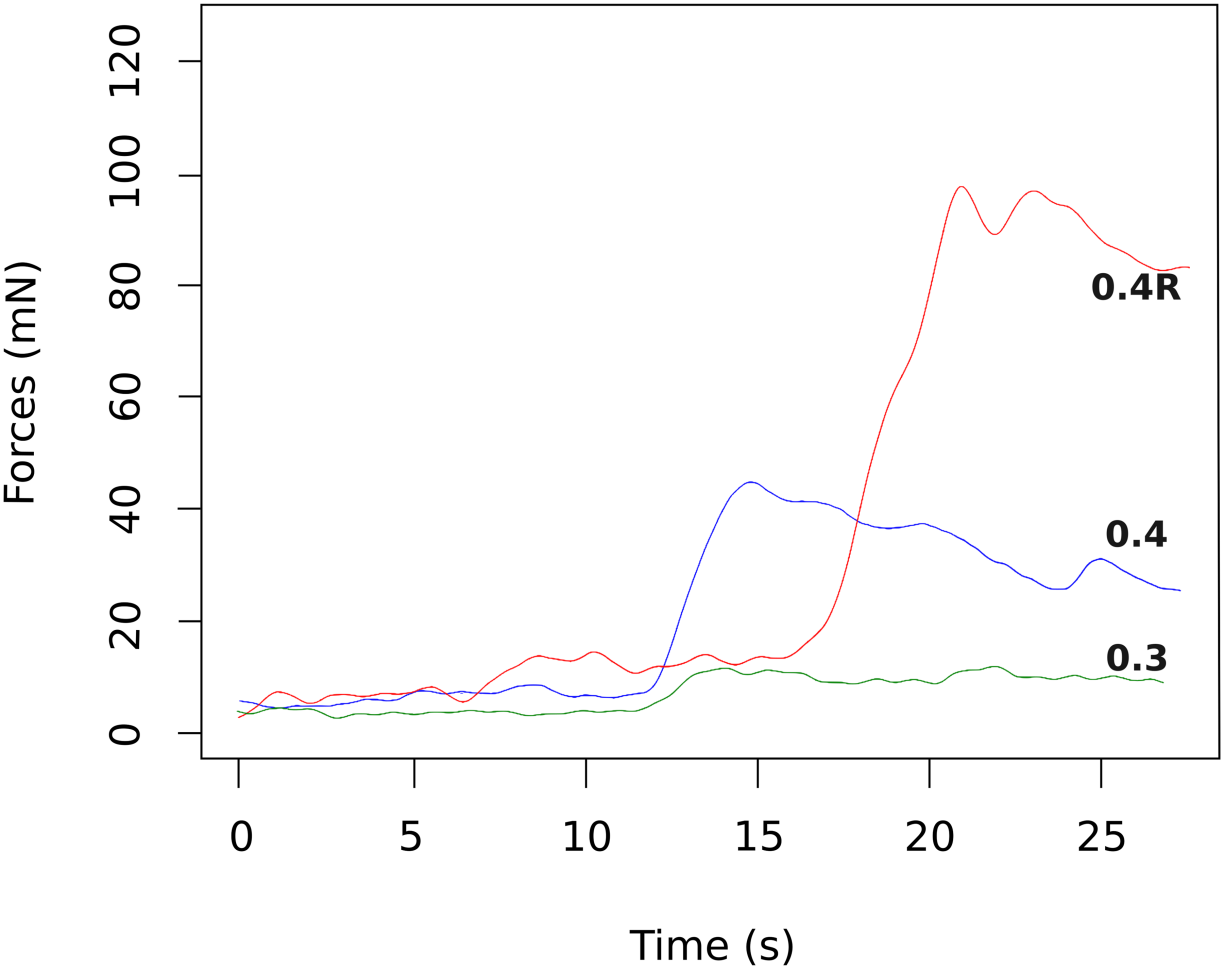
Array force insertions in mN (millinewtons) as function of insertion duration in a guinea pig model with three different array design. Insertion forces remained low in the first half on the insertion and then slowly rose and reached a maximum with the three array design at the end of the insertion. In this example, a higher peak force with the 0.4R arrays (red) can be observed compared 0.4(blue) and 0.3 (green) array design.

**Fig 6:**
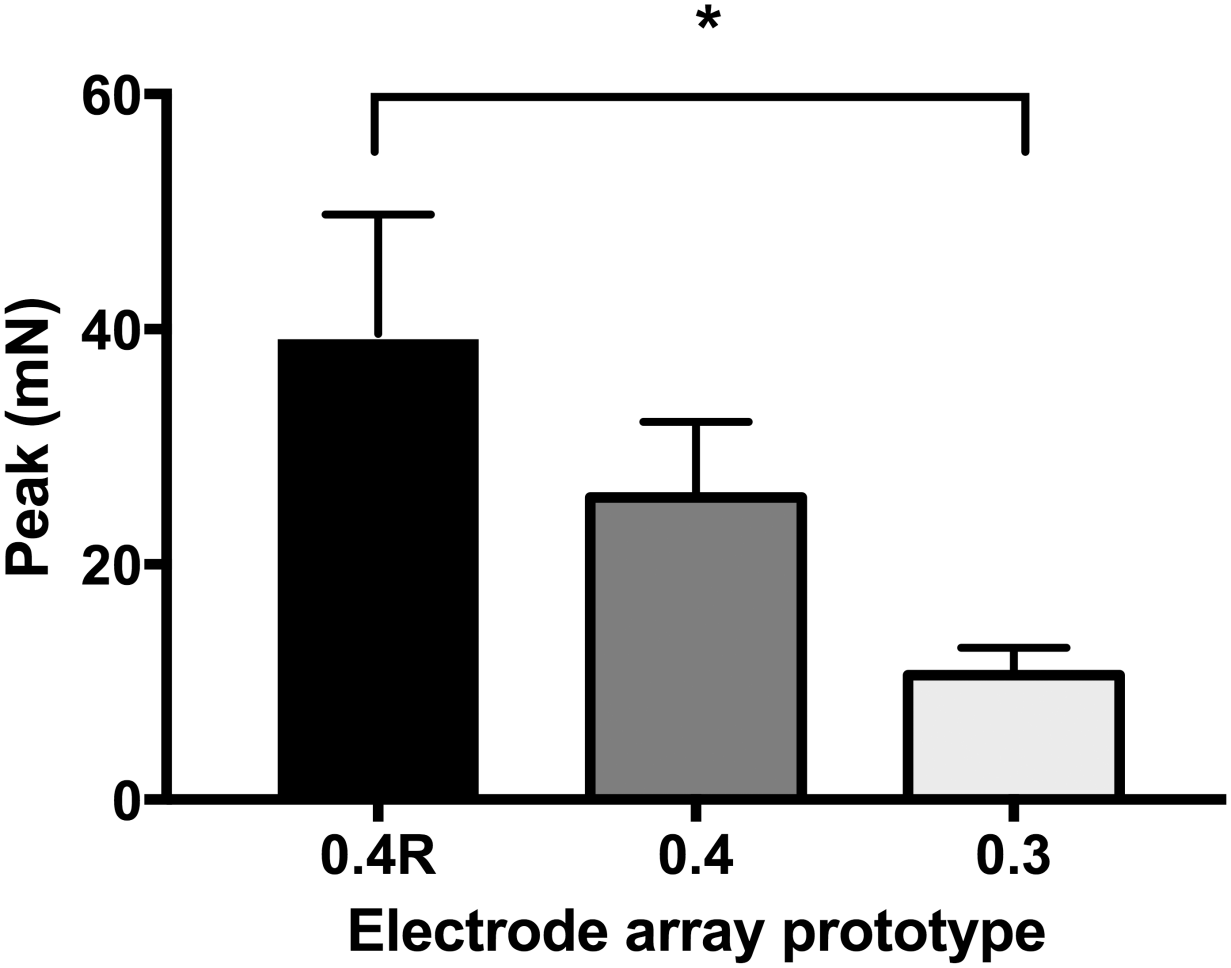
Maximal peak force measured during insertion of the three electrode array types. Electrode array geometry influences the maximal peak of the insertion force. Values are means± SEM. One-way Anova, *p<0.05.

**Fig 7:**
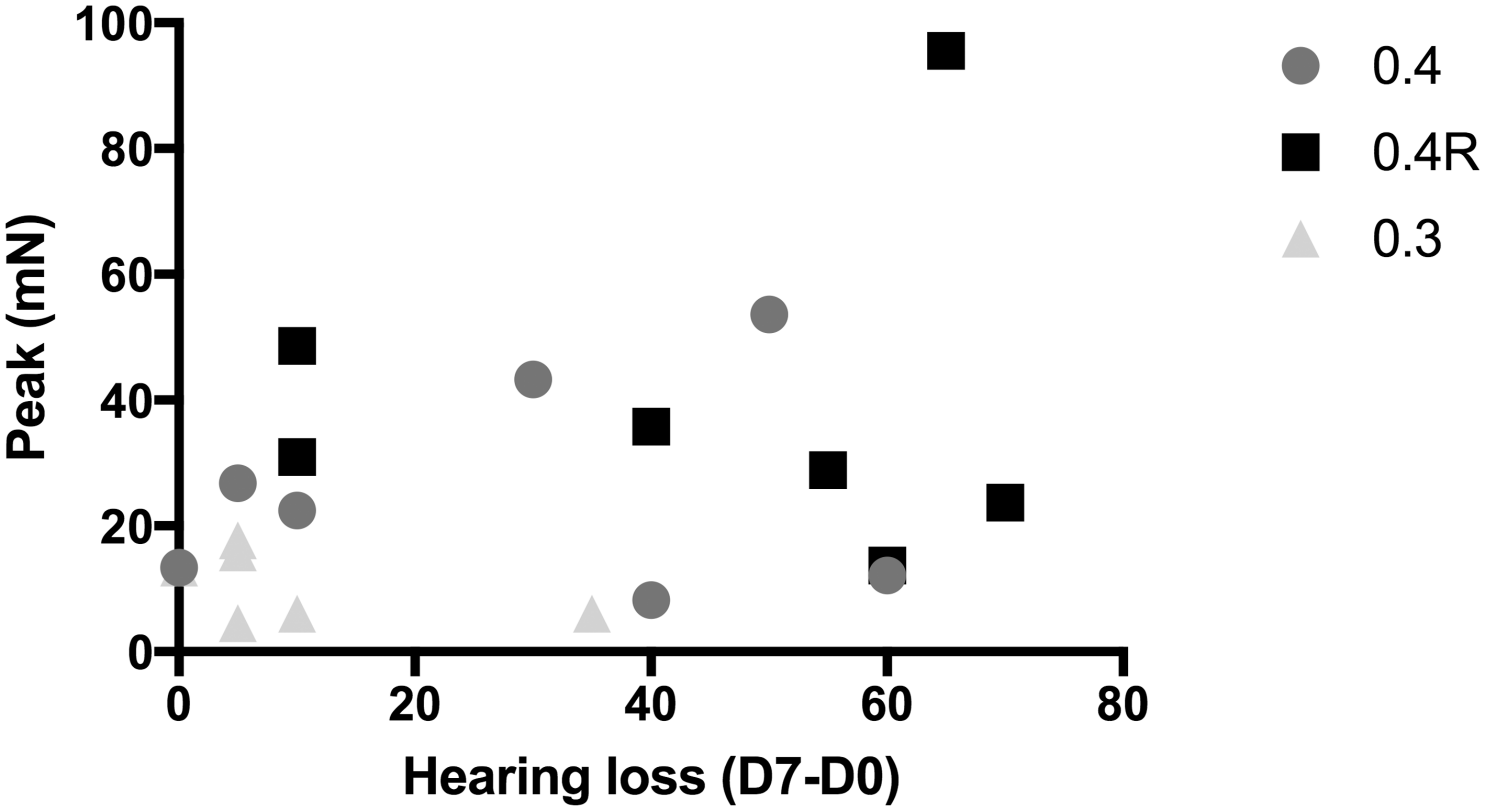
Correlation between hearing loss (dB) at 16000 Hz on D7 and maximal peak force measured during insertion of the three types of electrode array. 0.4R (black squares) (n = 7), 0.4 (dark grey circles) (n = 7), and 0.3 (light grey triangles) (n = 6) electrode arrays. Linear regression, respectively: Y = 0.31X + 17.2, Y (mN), X (dB), n = 20, R = 0.13, slope not different from 0, p = 0.12.

No significant difference was observed for jerk or momentum between the three electrode array types. Yet, the maximal peak of insertion force was higher with the 0.4R array than with the 0.3 array (56 ± 23.8 mN, n = 7 vs 26 ± 8.7 mN, n = 6, two-way Anova, p<0.05) (Figs 5 and 6). Nevertheless, the maximal peak of insertion force was not correlated with hearing loss after implantation (Fig 7).

### Position of the electrode array in the cochlea

For this part of the study, 12 of the 20 guinea pigs were selected: six animals had good hearing with a minimal threshold shift (less than 10 dB threshold shift compared to pre-operative hearing), and six animals had poor hearing with a threshold shift over 40 dB at 16000 Hz on D30 compared to pre-implantation hearing. Among the 12 animals, 7 had a 0.4 mm array (standard array, relative stiffness 1), 2 had a 0.4R array (stiff array, relative stiffness 6.8) and 3 had a 0.3 mm array (relative stiffness 0.8).

For the six guinea pigs with poor hearing (Table 2):

- Three had an incorrectly positioned electrode array, one was translocated into the *scala media* and two were in the A position according to our classification scheme (one in contact with the basilar membrane and one in contact with fractured osseous spiral lamina) (Fig 1B).
- Two animals had a well-positioned electrode array but with fibrosis around it, reaching the basilar membrane (more than 80% of the basal turn was invaded by fibrosis) (n = 2) (Fig 1C).
- One animal had an incorrectly positioned electrode array (A position) surrounded by fibrosis (between 10% and 50% of the basal turn was invaded by fibrosis).

**Table 2:** Hearing loss (dB) at 8000 and 16000 Hz, between D7 and D0, between D30 and D0, and between D30 and D7, in the 6 guinea pigs studied for macroscopic anatomy and with poor hearing (hearing loss > 40 dB).

Animal and array type	Macroscopic anatomy findings	Eshraghi grading system [12]	Hearing loss (dB)					
			D7-D0		D30-D0		D30-D7	
			8000	16000	8000	16000	8000	16000
GP1, 0.3	Fract. OSL	4	20	60	30	55	10	-5
GP2, 0.4	Translocation	3	50	65	55	55	5	-10
GP3, 0.4	BM Contact	1	70	70	70	75	0	5
GP4, 0.4	BM Contact + Fibrosis	1	35	60	60	70	25	10
GP5, 0.4R	Fibrosis only	0	15	60	35	65	20	5
GP6, 0.4	Fibrosis only	0	30	35	35	50	5	15

Individual hearing loss values measured at 8 and 16 kHz in the 6 guinea pigs (GP) with poor hearing and studied for macroscopic anatomy.

Abbreviations: Fract. OSL, position with fractured osseous spiral lamina; Translocation, translocation to the scala vestibuli; BM Contact, position with basilar membrane contact; BM Contact + Fibrosis, position with basilar membrane contact associated with fibrosis.

In the poor hearing group at D30, animals with incorrect position of the electrode array (n = 4) had 44 ±18.5 dB and 64 ± 4.1 dB hearing loss at 8000 and 16000 Hz, respectively on D7. In the poor hearing group at D30, animals with correct position of the electrode array (n = 2) had 15 and 30 dB hearing loss at 8000 Hz on D7, and 60 and 35 dB hearing loss at 16000 Hz on D7.

In the poor hearing group at D30, two patterns of evolution of hearing loss could be observed between D7 and D30. First, animals with fibrosis around the electrode array (n = 3, 15%) suffered a decrease in hearing between D7 and D30 (16 ± 8.5 dB at 8000 Hz, 10 ± 4.1 dB at 16000 Hz). Second, animals without fibrosis around the array (n = 3), showed stable hearing between D7 and D30 (5 ± 4.1 dB at 8000 Hz, -3.3 ± 6.2 dB at 16000 Hz).

None of the six guinea pigs with good hearing had either translocated or incorrectly positioned arrays or fibrosis around the array (less than 10% fibrosis in the basal turn) (Fig 1D).

## Discussion

In the normal hearing guinea pig, cochlear implantation using a thin electrode array (0.3) was less traumatic at high frequencies than implantation with a larger and thicker array (0.4R). Moreover, at D7, the 0.4 electrode array was also less traumatic at 16000 Hz than the 0.4R electrode array. Furthermore, the maximal peak of the insertion force was higher with the 0.4R electrode array than with the 0.3 electrode array. However, no correlation was observed between the insertion force and post-implantation hearing loss. From studies on macroscopic anatomy, a hearing loss > 40 dB was associated with an incorrectly positioned electrode array and/or fibrosis around the electrode array.

### Choice and limitations of the methods used

Our protocol has been designed to maximize the reproducibility of the implantation (motorized insertion tool carried by a robotic arm), to reduce respiratory artifacts during insertion force measurements (hammock and frame), and to eliminate putative contralateral non-implanted cochlear ABR. Indeed, we have previously shown that, with a motorized insertion tool, the hearing of guinea pigs was better preserved than with manual insertion [9], and that frictional forces were lower [13,14], and there was less tremor responsible for jerk [8].

Considering the ABR measurements, we also chose to surgically deafen the contralateral side to be sure that our results were not affected by bone conduction as previously reported by Reiss *et al*. [15].

In addition, the area ratios (array/scala tympani) of the human cochlea and guinea pig cochlea are different. It has been estimated by Mamelle *et al*. that the area ratio was 32% at 180° in the guinea pig and 9% in the human cochlea with a 0.4 mm diameter array [9]. Thus, even though the study was designed to examine the trauma and preservation of residual hearing after implantation, the use of an animal model may limit interpretation of this study for clinical application.

### Correlation between hearing and electrode array type

Guinea pigs implanted with a stiffer electrode array (0.4R) had poorer hearing than those implanted with a thinner and more flexible electrode array (0.3). Considering the abundant clinical studies on this topic, this result was expected. Thus, in this animal model, we confirmed that thinner and more flexible electrode array insertions were more likely to preserve hearing.

It is reasonable to believe that both electrode array placement in the cochlea and fibrosis could influence residual hearing preservation outcomes after cochlear implantation. Mechanical trauma during insertion or a secondary decrease in the number of neurosensory cells by apoptosis or inflammation could lead to postoperative hearing loss [12,16]. Another hypothesis that may account for hearing loss after electrode array insertion is a venous return impairment [17].

Mechanical trauma during electrode array insertion can lead to elevation or rupture of the basilar membrane, electrode dislocation into the *scala vestibuli*, fracture of the osseous spiral lamina, or modiolus, or tearing of the stria vascularis as described by Eshraghi *et al*. [12]. This may lead to early postoperative hearing loss as observed in our study. Thus, in our macroscopic analysis, four out of 12 guinea pigs which had an initial trauma (contact with the basilar membrane n = 1, rupture of the basilar membrane n = 1, or osseous spiral lamina lesion n = 2) had a 64 ± 4.1 dB mean hearing loss at 16000 Hz on D7.

The guinea pig which had an electrode array in contact with the basilar membrane without any other lesions had a 75 dB loss at 16000 Hz on D30. One may assume that the basilar membrane vibration was dampened or blocked by the electrode array, thus impairing basilar membrane biomechanics and travelling wave propagation. Such a blocking mechanism by fibrosis was previously suspected in a mathematical model [18] and in animal studies [19,20].

After initial hearing loss, secondary hearing deterioration can be explained by inflammation or oxidative stress leading to fibrosis or osseous tissue formation. In the histology of an implanted patient postmortem, Nadol *et al*. described a fibrous scar in the cochlear basal turn [21]. This fibrosis was only present in the area of electrode array insertion and did not reach the cochlear apex. In our study, three animals had fibrosis around the electrode array. Among these animals, two had an electrode array which was correctly located in the *scala tympani*, with fibrosis which extended to the basilar membrane. The third animal had an electrode array in contact with the basilar membrane with fibrosis. In the O’Leary classification, fibrosis predominated on contact with the basilar membrane and lateral wall but not close to the modiolus [20]. The origin of this fibrosis is not well understood. It could be a fibrous scar secondary to foreign object reaction [21], a healing process secondary to a traumatic/hemorrhagic insertion [22,23] or an epithelial-to-mesenchymal transition [5]. Ryu *et al*. suspected that blood was responsible for the genesis of fibrosis because cochleae with blood injections had more extensive fibrosis almost reaching the cochlear apex with increased ABR threshold and decreased hair cells [24]. Furthermore, the fibrosis can lead to ossification lesions. Kamakura & Nadol highlighted a correlation between hearing performance, new bone formation and remaining ganglion spiral cells in human (11 post-implantation years) [23].

In previous animal models of cochlear implantation, postoperative fibrosis has also been studied and was observed in a higher percentage of animals compared to our results (15% of the animals in this study) [6,20,24]. One explanation could be that the macroscopic anatomy technique used in this study did not quantify the fibrosis as precisely as in other studies as it did not apply accurate specific histological criteria. But one could also assume that robot-based electrode array alignment and insertion with a motorized tool would lead to fewer anatomical lesions compared to manual insertion as it has previously been shown that motorized electrode array insertion can provide better hearing preservation compared to the manual technique [9].

In this study, we also found that guinea pigs implanted with 0.4R arrays had higher maximal peaks during force measurements than those implanted with the 0.3 array. However, no correlations between insertion force parameters (maximal peak of insertion force, momentum and jerk) and threshold shifts were seen at day 7 or day 30. Even though the animal was placed in a hammock and frame to limit artefactual respiratory movements, our force measurement set-up was not sufficiently accurate or sensitive to detect basilar membrane lesions during electrode array insertion. We measured the overall insertion force rather than measuring specific events such as basilar membrane rupture, spiral ligament tearing, or osseous spiral lamina breakage. Furthermore, it is probable that hearing loss is not just related to insertion force trauma but also to a combination of secondary factors following initial necrosis and apoptosis processes. To the best of our knowledge, there is no published data on a direct correlation between the insertion force of a cochlear implant array (with accurate reported values) and hearing preservation. This was one of the objectives of our study design but we did not obtain sufficiently robust data to draw firm conclusions. Either we did not measure the force profile accurately enough or we did not analyze the data with appropriate metrics, or perhaps hearing preservation is governed by other factors (e.g. fibrosis, basilar membrane micromechanics, impairments, etc.).

## Conclusion

Cochlear implantation with a motorized insertion tool carried by a robotic arm, at a controlled speed, associated with a 0.3 mm external diameter electrode array, could limit post-electrode array insertion hearing loss to 15 dB, in guinea pig. Moreover, in this animal model, electrode parameters (stiffness and external diameter) influenced insertion forces, and also affected hearing loss. Hearing loss after implantation was related to electrode array position in the cochleae and fibrosis in the *scala tympani*.

These results motivate further improvement in electrode design and insertion techniques to reduce postoperative fibrosis, and enhance electrode array positioning avoiding contact with the basilar membrane to improve residual hearing preservation after cochlear implantation.
